# Head and Neck Tumor Segmentation Using Pre-RT MRI Scans and Cascaded DualUNet

**DOI:** 10.1007/978-3-031-83274-1_14

**Published:** 2025-03-03

**Authors:** Mikko Saukkoriipi, Jaakko Sahlsten, Joel Jaskari, Ahmed Al-Tahmeesschi, Laura Ruotsalainen, Kimmo Kaski

**Affiliations:** 1 Department of Computer Science, Aalto University, Espoo, Finland; 2 Department of Computer Science, University of Helsinki, Helsinki, Finland; 3 Department of Electronic Engineering, University of York, York, UK; 4 The Alan Turing Institute, London, UK

**Keywords:** Cascaded deep neural networks, Dual-stage refinement, 3D UNet, HNTS-MRG, MRI Head and Neck Tumor Segmentation

## Abstract

Accurate segmentation of the primary gross tumor volumes and metastatic lymph nodes in head and neck cancer is crucial for radiotherapy but remains challenging due to high interobserver variability, highlighting a need for an effective auto-segmentation tool. Tumor delineation is used throughout radiotherapy for treatment planning, initially for pre-radiotherapy (pre-RT) MRI scans followed-up by mid-radiotherapy (mid-RT) during the treatment. For the pre-RT task, we propose a dual-stage 3D UNet approach using cascaded neural networks for progressive accuracy refinement. The first-stage models produce an initial binary segmentation, which is then refined with an ensemble of second-stage models for a multiclass segmentation. In Head and Neck Tumor Segmentation for MR-Guided Applications (HNTS-MRG) 2024 Task 1, we utilize a dataset consisting of pre-RT and mid-RT T2-weighted MRI scans. The method is trained using 5-fold cross-validation and evaluated as an ensemble of five coarse models and ten refinement models. Our approach (team FinoxyAI) achieves a mean aggregated Dice similarity coefficient of 0.737 on the test set. Moreover, with this metric, our dual-stage approach highlights consistent improvement in segmentation performance across all folds compared to a single-stage segmentation method.

## 1 Introduction

Radiation therapy (RT) remains a cornerstone in the treatment of head and neck cancer (HNC), including oropharyngeal cancer (OPC). RT requires accurate tumor delineation for effective treatment outcomes while minimizing damage to surrounding healthy tissue. This requirement is particularly acute in HNC due to close proximity of tumors to critical and radiation sensitive structures, such as neural and optic pathways [1]. However, accurately delineating gross primary tumor volumes (GTVp) and metastatic lymph nodes (GTVn) in HNC is challenging, as evidenced by the high interobserver variability among oncologists [1].

In order to address these challenges, various imaging modalities have been explored for their ability to provide distinct tissue and functional contrasts. The primary imaging modalities for HNC detection are computed tomography (CT), positron emission tomography (PET), and magnetic resonance imaging (MRI) [1]. CT has traditionally been preferred for its spatial accuracy, but it suffers from scatter artifacts, especially from dental amalgam, and difficulties to differentiate tumors from adjacent glottic musculature [1]. This leads to high interobserver variability when using CT alone [2]. To improve tumor delineation, PET has been integrated with CT as it provides metabolic information that aids in differentiating tumor cells from healthy tissue due to their elevated metabolic activity. Despite its low spatial resolution and partial volume effects, combining PET with CT or MRI enhances the accuracy of tumor delineation [1].

We note that MRI offers superior spatial resolution and soft tissue contrast, with the added benefit of not exposing patients to radiation, which provide notable advantage over both CT and PET [3]. This capability enhances the differentiation of adjacent soft tissues and allows for precise delineation of tumor margins. This is crucial for mapping deep tissue invasion, detecting small tumors, and characterizing complex tumor structures [4]. The improved contrast resolution also enables more sensitive evaluation of perineural invasion, intracranial extension, vascular infiltration, and bone marrow involvement [4]. However, MRI has also some limitations compared to CT. The procedure is considerably more time-consuming and expensive, which limit its use as a first-line imaging modality. In addition, patients may find it difficult to remain still for the extended duration of the scan [4].

Artificial intelligence (AI) tools have shown potential to assist radiologists in enhancing diagnostic quality while reducing their workload [5]. Although there is substantial research on automatic segmentation of oropharyngeal cancer in cases of multimodal PET-CT [1,6–9] and PET-MRI [1,6], the segmentation of HNC from MRI scans alone has received comparatively less attention [10]. Recent studies have explored various approaches to segmenting HNC from MRI scans. Schouten et al. [11] investigated the use of T1-weighted, T1gad, and STIR MRI modalities for HNC segmentation, reporting an average Dice similarity coefficient (DSC) of 0.49. Liedes et al. [12] found that a 2D U-Net struggled to segment HNC using only T1 SPIR, T1 TSE, or T2 TSE MRI images, but observed considerable improvement when these MRI modalities were combined with PET images. Wahid et al. [10] compared various MRI modalities and observed that T2-weighted (T2w) MRI alone achieved an average DSC of 0.72, which was slightly improved by incorporating additional modalities. These findings highlight ongoing efforts to enhance accuracy of HNC segmentation from MRI scans.

Regulatory and privacy constraints require local data processing at hospitals, which may limit the use of powerful cloud computing resources [13]. While real-time tumor delineation is essential in procedures such as MR-Linac, pre-RT setups generally operate without this requirement. Therefore, automatic tumor delineation in this case does not need to take place in real time, thus allowing more processing time compared to many other computer vision tasks.

Previous works have primarily focused on 2D or 3D UNet variants for OPC GTVp and GTVn segmentation. However, the potential benefits of cascaded deep learning have not yet been explored for this task. Cascading involves a sequential architecture in which later stages refine the outputs of earlier ones, often by progressively utilizing more detailed features [14]. This can be achieved either within a single network or through training multiple networks in stages. Cascade networks that segment the region of interest from progressively increasing MRI scan resolution have been studied for brain tumor segmentation [15,16] and organs-at-risk segmentation [17]. There have also been studies that utilize the cascading approach in the full resolution for all the stages, e.g., in bladder cancer segmentation [18] and brain tumor segmentation [19,20] in MRI as well as fetal head and abdomen segmentation in ultrasound images [21].

Inspired by prior work in cascaded deep learning segmentation, we propose a two-stage cascaded ensemble for the segmentation of the OPC GTVp and GTVn using T2w MRI scans. Our approach decomposes the task into two distinct subtasks, both performed by a deep learning model which are trained and evaluated separately. In the first-stage, a model performs binary segmentation to distinguish tumors from healthy tissue, while the second-stage model classifies the tumor regions into the two classes while refining the segmentation boundaries. This sequential approach maintains memory efficiency equivalent to a single model with a cost of additional computational time. Moreover, separating the tasks enables independent evaluation of segmentation and classification performance, providing insights into model-specific errors.

## 2 Methods

In this section, we describe the dataset and its preprocessing as well as present our DualUNet approach in terms of architecture, model training, and evaluation.

### 2.1 Datasets

The HNTS-MRG 2024 training dataset comprises both fat-suppressed and non-fat-suppressed T2-weighted MRI scans of 150 head and neck cancer patients imaged at MD Andreson Cancer Center (MDACC). Patient data includes pre-RT scans e.g., imaged 1–3 weeks before radiotherapy and mid-RT scans e.g., imaged 2–4 weeks during radiotherapy. Most of the patients are diagnosed having oropharyngeal cancer and the rest have cancer of unknown primary. Each scan has been segmented for primary gross tumor volumes (GTVp) and metastatic lymph nodes (GTVn) by 3–4 physicians independently and verified by senior radiation oncology faculty. The final ground truth segmentation is provided by simultaneous truth and performance level estimation algorithm (STAPLE) [22].

The scans exhibit considerable variability in both imaging resolution and spatial dimensions. Analysis of the training dataset determined the voxel resolutions to be approximately [0.4–1.0] mm × [0.4–1.0] mm × [1.0–2.5] mm spacing and [512–768] × [480–768] × [64–176] dimension.

### 2.2 Cascaded Segmentation Framework

#### DualUnet.

**Fig. 1.**
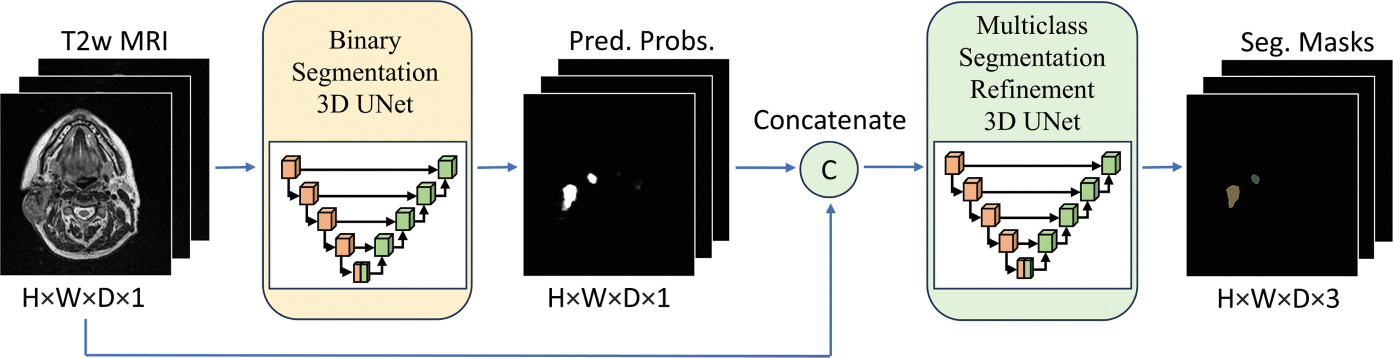
DualUNet architecture consisting of a binary and a multiclass refinement 3D UNet segmentation models. The input of the refinement UNet is the concatenation of T2-weighted (T2w) MRI and the sigmoid output of the binary UNet.

The proposed cascaded UNet framework named *DualUNet*, is illustrated in Fig. 1, consists of two individual models, both based on residual UNet [23] due to its reputation for accuracy and robustness in medical imaging tasks. The first-stage UNet uses T2w MRI scans as input and produces a single output channel with sigmoid non-linearity applied separating background and any tumor segmentations. The second-stage UNet functions as a refinement model and it uses the original T2w MRI scans and the output from the first-stage UNet as inputs and produces multiclass outputs for background, GTVp, and GTVn with softmax non-linearity.

#### UNet.

Both of the UNet implementations in the framework feature a progressive increase in channel counts for each of the blocks, i.e., 32, 64, 128, 256, and 512, throughout the encoder, enabling detailed feature extraction across multiple scales. A stride pattern of 1, 2, 2, and 2 is employed within each block, starting with a stride of 1 to preserve high-resolution details and increasing to stride 2 in deeper layers, which broadens the receptive field and reduces computational requirements. Each block includes two convolutional layers and a residual connection, the latter of which is used to mitigate the vanishing gradient problem and ensure smooth gradient flow during training. Each convolution layer is followed by Instance Normalization layer to standardize features across each channel and a PReLU non-linearity [24].

The method was implemented with PyTorch 2.2.2 and MONAI 1.3.0 [25], utilizing a 3D UNet model with residual connections [23].

### 2.3 Training Procedure

#### Preprocessing.

We utilized the HNTS-MRG2024 dataset consisting of 150 head and neck cancer patients with segmented targets. We developed the method using 5-fold cross-validation based on the patients. The training folds included both pre-RT and mid-RT scans of the patients and the validation fold included only pre-RT scans.

In accordance with prior research on tumor segmentation from T2w MRI images [10,26], non-zero voxels were standardized using Z-score normalization (mean = 0, standard deviation = 1) that improves the dynamic range of clinically significant regions and improves numerical stability.

Given the memory constraints of the HNTS-MRG 2024 competition, particularly the Nvidia T4 GPU’s 16 GB limit and the variability in volume sizes (up to 768 × 768 × 176 voxels), we implemented cropping during inference and training to avoid exceeding GPU memory. For both the training and testing phases, we used a fixed patch size of 408 × 408 × 64 voxels. In training the first-stage models, we applied the probabilistic crop sampling technique proposed by Myronenko et al. [27], wherein the crop center is selected based on ground truth labels, with a probability distribution of 45% for GTVp, 45% for GTVn, and 10% for background. In contrast, for the second-stage models, random cropping was employed, as the probabilistic approach did not demonstrate improvements in performance. Model inference during the evaluation phase was carried out using a sliding window method, maintaining the same patch size and averaging the results over a 50% overlap.

#### Data Augmentation.

In order to ensure model performance with heterogeneous volumetric medical imaging data, we use a wide range of augmentation methods consisting of spatial and voxel intensity value changes. The spatial augmentations consisted of random mirroring independently on each axis with a probability of 10% as well as random rotation of up to 30 °C and translations within a range of 16 pixels, both with 50% probability. Moreover, the variation in scan resolution needed to be accounted as we used native resolution scans without resampling. During training, we employed a custom resampling augmentation method to cover all resolution ranges identified in the training dataset. The custom resampling method was applied with 50% probability on each axis and with target resolution based on uniform distribution of the training dataset resolution ranges. In terms of intensity augmentations, we used contrast adjustment with gamma range 0.5 to 1.5, intensity shifting with 10% offset, random Gaussian noise with standard deviation of 0.1, and Gaussian blurring, all with 25% probability.

#### Loss.

In our binary segmentation model, we employ a combined Dice loss and binary Cross-Entropy (BCE) loss. For the multiclass refinement model, we utilize a combination of Dice loss and Cross-Entropy (CE) loss. These combined losses have been shown to be an effective strategy for head and neck cancer segmentation from PET-CT images [27,28]. The composite loss functions, with uniform weight of 1, are formulated as follows:

(1) $${ℒ}_{\text{DiceBCE}}={ℒ}_{\text{Dice}}+{ℒ}_{BCE},$$

(2) $${ℒ}_{\text{DiceCE}}={ℒ}_{\text{Dice}}+{ℒ}_{CE},$$

where Dice Loss is denoted by ${ℒ}_{\text{Dice}}$, Binary Cross-Entropy loss by ${ℒ}_{BCE}$ and Cross entropy by ${ℒ}_{\text{CE}}$. The Dice Loss is defined as follows:

(3) $${ℒ}_{\text{Dice}}=1-\frac{2\sum_{i}\;p_{i}g_{i}\;+\;\epsilon }{\sum_{i}\;p_{i}\;+\;\sum_{i}\;g_{i}\;+\;\epsilon },$$

where p and g represent the model output and ground truth segmentation, respectively, and $\epsilon =1\times 10^{-5}$ is a smoothing factor to prevent division by zero. The BCE and CE Losses are defined as:

(4) $${ℒ}_{BCE}=-\frac{1}{N}\sum_{i}^{N}\;\left[g_{i}\log\left(p_{i}\right)+\left(1-g_{i}\right)\log\left(1-p_{i}\right)\right],$$

(5) $${ℒ}_{CE}=-\frac{1}{N}\sum_{i}^{N}\;\sum_{c=1}^{C}\;g_{i,c}log\left(p_{i,c}\right),$$

where $p_{i}$ and $g_{i}$ are the predicted probability and ground truth for pixel i, respectively, and N is the number of pixels. For the multiclass case, C denotes the number of classes, with $p_{i,c}$ and $g_{i,c}$ representing the predicted probability and ground truth for class c at pixel i. These composite loss functions facilitate comprehensive optimization of deep learning segmentation by addressing both the overlap of imbalanced foreground classes and per-pixel classification accuracy [29].

#### Optimization.

Model parameters are optimized using AdamW optimizer with an initial learning rate of 2 × 10^−4^, which is decreased to zero at the end of the final epoch using a cosine annealing scheduler. All models are trained for 300 epochs with a mini-batch size of 1 on a single 80 GB NVIDIA A100 machine. Additionally, we implement weight decay regularization set to 1 × 10^−5^ and use dropout regularization with a probability of 10%.

The first-stage and second-stage models are trained separately. For first-stage model training, we combine the GTVp and GTVn classes, using this as a binary target. For the second-stage model training, the first-stage model generates sigmoid probability volumes, which are then used as an input alongside the original volume. Prior research suggests that such refinement models may become overly reliant on the first-stage segmentation [28]. To mitigate this, we randomly drop the first-stage segmentation input with a probability of 10%.

In addition, the training convergence is improved by leveraging model weight pretraining on the HECKTOR 2022 dataset for OPC GTVp and GTVn segmentation using PET-CT scans [9].

### 2.4 Model Validation

We evaluate the segmentation performance using class-wise mean of the Aggregated Dice Similarity Coefficient (${DSC}_{\text{agg,mean}}$) which is defined as follows:

$$V_{c,p}=\sum_{i}\;\left[Y_{i,p}=c\right],$$

$$P_{c,p}=\sum_{i}\;[arg\;\underset{j}{max}\;{\overset{ˆ}{Y}}_{j,p}=c],$$

$${TP}_{c,p}=\sum_{i}\;[Y_{i,p}=c]\cdot \left[\underset{j}{\arg max}{\overset{ˆ}{Y}}_{j,p}=c\right],$$

(6) $${DSC}_{agg,c}=2\frac{\sum_{p}\;{TP}_{c,p}}{\sum_{p}\;\left(V_{c,p}\;+\;P_{c,p}\right)},$$

(7) $${DSC}_{\text{agg,mean}}=\frac{1}{2}\left({DSC}_{agg,GTVp}+{DSC}_{agg,GTVn}\right),$$

where $V_{c,p}$ and $P_{c,p}$ are the sum of labelled and predicted voxels i and j, respectively, for class c of patient $p,\;{TP}_{c,p}$ is the sum of correctly predicted voxels for class c of patient p, and ${DSC}_{agg,c}$ is the aggregated Dice similarity coefficient for class c. Unlike the conventional Dice similarity coefficient in which multiclass segmentation results are averaged with equal weighting on each class, the ${DSC}_{agg}$ offers more robust metric, in terms of individual misclassifications, for the overall dataset segmentation performance.

Models were trained and validated using a 5-fold cross-validation approach. The 150 patients in the training dataset were randomly partitioned into five non-overlapping subsets, each containing 30 patients. Each subset served as the validation set, while the remaining 120 patients were used for training. Only pre-RT scans were used during validation to align with the HNTS-MRG 2024 Task 1 focus on pre-RT segmentation.

**Fig. 2.**
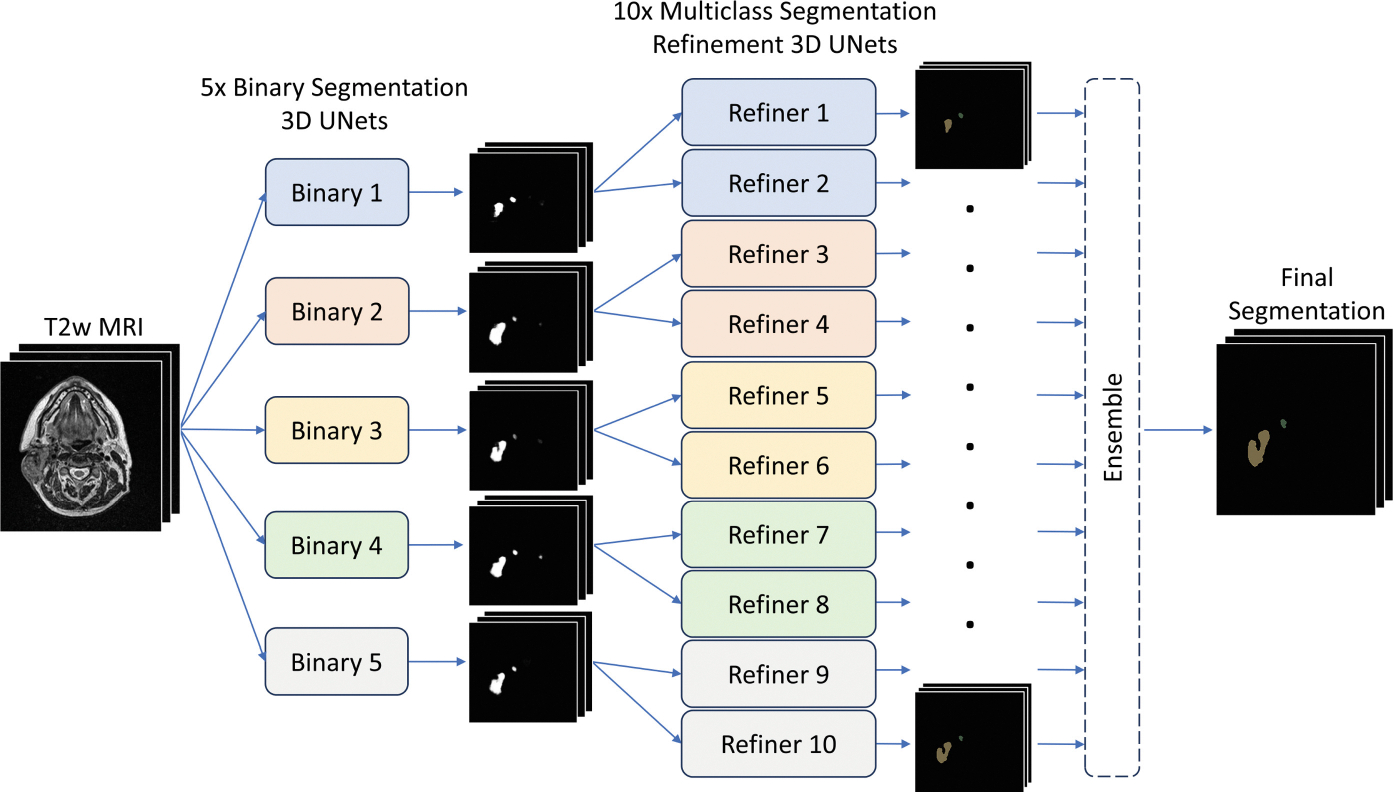
Test time cascade ensemble with five coarse models and ten refinement models. The five fold used for the five binary and ten refiner model training are colored separately.

In the test set performance evaluation, we employ an ensemble consisting of five first-stage binary models and ten second-stage multiclass models. In each fold one first-stage model and two second-stage models were used. This approach results in ten outputs which are averaged and followed by argmax-operation for the final segmentation output. The test time ensemble approach is illustrated in Fig. 2.

## 3 Results

On the separate test dataset with (N=50), the DualUNet turned out to have ${DSC}_{\text{agg,mean}}$ value of 0.737. The class specific performances were 0.697 for DSC_agg_,_GTVp_ and 0.777 for DSC_agg_,_GTVn_. The 50 test patients were evaluated through the HNTS-MRG 2024 competition official evaluation tool at the Grand-challenge.org platform.

In terms of the overall performance on training dataset, the DualUNet outperformed UNet across all folds with lowest performance on Fold 4 with 0.679 ${DSC}_{\text{agg,mean}}$ and highest performance in Fold 3 with 0.736 ${DSC}_{\text{agg,mean}}$. Specifically, the lowest difference between the methods was with Fold 2 with 2.2% and largest difference with Fold 4 with 8.0% improved performance with DualUNet. The full comparison is shown in Table 1.

When considering only the primary gross tumor volume segmentation on training dataset, the DualUNet outperformed the UNet across all folds with an average increase of 5.4% in DSC_agg,GTVp_ on the full dataset. In terms of the gross tumor volume of nodal disease, DualUNet outperformed UNet in four out of five folds with the average increase of 4.7% in DSC_agg,GTVn_ using the full dataset. Specifically, the largest difference was with Fold 4 in which DualUNet had a value of 0.818 DSC_agg_,_GTVn_ while UNet had a value of 0.758 DSC_agg_,_GTVn_. The lowest difference was with Fold 2, where DualUNet had a value of 0.805 DSC_agg,GTVn_ while UNet had a value of 0.812 DSC_agg,GTVn_.

## 4 Discussion

In this study, we proposed and evaluated a dual-stage 3D UNet architecture to detect and segment primary gross tumor volumes and metastatic lymph nodes in the head and neck area using MRI scans. Our approach employs a cascaded deep neural network with a dual-stage UNet architecture, where the first-stage produces binary segmentations, and the second-stage refines the segmentation and classifies the regions into multiclass segmentations. The performance of the DualUnet method was assessed in the HNTS-MRG 2024 challenge pre-RT segmentation task, yielding a ${DSC}_{\text{agg,mean}}$ of 0.757 with 5-fold cross-validation, a 4.9% improvement in ${DSC}_{\text{agg,mean}}$ over the standard UNet, and a ${DSC}_{\text{agg,mean}}$ of 0.737 on the test set. This study underscores the advantages of cascaded dual-stage deep neural networks for tasks where inference time is less critical. Compared to UNet, DualUNet employs two separate UNets in a serial manner, approximately doubling the inference time.

Interestingly, segmentation performance was generally higher for GTVn than for GTVp. This is in contrast with previous studies using deep learning to segment these tissue types from PET-CT scans, where the performance on GTVp has been generally better [9]. Additional analysis is required to determine whether this is due to difference in scanning modalities, deep learning methods, dataset characteristics, or other factors.

A key strength of our approach is its ability to manage complex segmentation tasks by decomposing them into binary and multiclass subtasks. This separation enables more targeted refinement of the segmentation process and provides clearer insights into the performance of each stage. The performance of the approach on the test set underscores its effectiveness in addressing the variability in segmentation quality typically observed in clinical settings.

Variability in image dimensions and resolution presents two strategies i.e., scaling images to a selected standard resolution such as 1mm^3^ [27] or maintaining the native resolution that may need to be accounted for. In this study, we retained the native resolution and employed volume resizing augmentation to enable the trained model to accommodate a range of resolutions identified in the training dataset. In this approach, the proposed resampling augmentation was observed to have a significant impact on model performance. However, comparing these two strategies falls outside the scope of this work and is left for future investigation. As a limitation, the variable resolution strategy may have low performance with rare resolutions that were underrepresented during training. In order to ensure good performance across all resolutions, it is essential to use sufficient resolution augmentation during training and include rare resolutions in the model validation.

Based on our initial tests, the inclusion of mid-RT scans in addition to preRT scans in the training data improved the results. Although, the mid-RT scans have distinct properties, we hypothesize that the additional data outweighs the negative effects. However, this analysis is left for future work.

Overall, our dual-stage UNet approach represents a considerable advancement in automated HNC segmentation and may provide a promising tool for clinical practice pending clinical validation. The robust performance of the method and its adaptability to the complexities of clinical data highlight the potential for improving tumor delineation and treatment planning in radiotherapy.

## 5 Conclusion

In summary, we have demonstrated that our novel dual-stage cascading 3D UNet approach for HNC segmentation results in notable improvements in the segmentation accuracy and explainability in contrast to single stage approach. These findings underscore the potential of this approach to improve tumor delineation and refine treatment planning in radiotherapy.

## Figures and Tables

**Table 1.** Comparison of UNet and DualUNet performance on the validation folds of the training dataset (N = 150). Results are provided per fold (N = 30) and for the full set. Percentage changes relative to UNet are shown, with increases indicated by green arrows up and decreases by red arrows down.

Metric	Model	Fold 1	Fold 2	Fold 3	Fold 4	Fold 5	Full Set

DSC_agg, GTVp_	UNet	0.706	0.668	0.729	0.620	0.644	0.677
	DualUNet	0.726	0.709	0.736	0.679	0.720	0.716
		↑ 2.8%	↑ 5.8%	↑ 1.0%	↑ 8.7%	↑ 10.6%	↑ 5.4%

DSC_agg, GTVn_	UNet	0.773	0.812	0.685	0.758	0.762	0.758
	DualUNet	0.802	0.805	0.763	0.818	0.793	0.795
		↑ 3.6%	↓ 0.9%	↑ 10.2%	↑ 7.3%	↑ 3.9%	↑ 4.7%

DSC_agg, mean_	UNet	0.740	0.740	0.707	0.689	0.703	0.718
	DualUNet	0.764	0.757	0.750	0.749	0.757	0.755
		↑ 3.1%	↑ 2.2%	↑ 5.7%	↑ 8.0%	↑ 7.1%	↑ 4.9%
